# Effect of Elicitor Treatments on Quality Attributes in Blueberry: Implications of Cultivar and Environmental Conditions

**DOI:** 10.3390/plants13081105

**Published:** 2024-04-15

**Authors:** Gabriele Cola, Beatrice Cavenago, Claudio Sebastiano Gardana, Anna Spinardi

**Affiliations:** 1 Department of Agricultural and Environmental Sciences (DISAA), Università Degli Studi Di Milano, 20133 Milan, Italy; gabriele.cola@unimi.it (G.C.); beatrice.cavenago@unimi.it (B.C.); 2 Department of Food, Environmental and Nutritional Sciences (DeFENS), Università Degli Studi Di Milano, 20133 Milan, Italy; claudio.gardana@unimi.it

**Keywords:** chitosan, melatonin, southern highbush, northern highbush, phenolic compounds

## Abstract

Elicitors of plant defence responses can trigger defence mechanisms that are able to protect plant tissues from biotic or abiotic stresses. Since one defence response involves the activation of secondary metabolites’ biosynthesis, the purpose of this study was to evaluate the effect of chitosan and melatonin pre-harvest treatments on the quality and the nutritional parameters of the fruits of blueberry (*Vaccinium corymbosum* L.). Across the two years of experiment, three different cultivars (cv.s. ‘Cosmopolitan’, ‘Hortblue Poppins’ and ‘Legacy’) were treated with 1% chitosan or 100 µM melatonin every two weeks during the ripening season and ripe fruits were progressively harvested and analysed. The treatment with both elicitors had only slight effects on dry matter, soluble solids content, titratable acidity and pH, with a cultivar-dependent response. On the other hand, elicitors significantly affected the levels of phenylpropanoid and antioxidant compounds in all cvs. in both years, with a higher accumulation of total anthocyanins and phenolics and the enhancement of the antioxidant capacity, with positive effects on the nutraceutical quality of fruits. The anthocyanin profile in terms of both absolute concentrations and the relative proportion of single anthocyanins was affected by both harvest year and cv., highlighting the role of the genetic background in the plant response to environmental conditions (with particular reference to summer heat stress) and to elicitor treatments.

## 1. Introduction

Plant disease resistance is dependent on both pre-existing physical and chemical barriers associated with plant-innate immunity and plant-induced immunity, where signalling molecules activate the defence response to protect plant tissues from damage caused by biotic or abiotic stresses [1,2]. In the absence of any pathogen attack, these defence mechanisms may be induced by physical or chemical elicitation [3,4], resulting in enhanced resistance to pathogens and diseases and tolerance to abiotic stress and adversity. In fact, defence priming supports a faster, stronger and more sustained response to stresses [5,6,7].

From a sustainability point of view, induced resistance also has a positive impact on the environment due to the absence of toxic residues on the plant, on the fruits and in the environment. Furthermore, the induced resistance reduces the application of pesticides.

Regarding the time of application, when compared to postharvest approaches, preharvest elicitor treatments are expected to favour a better-coordinated allocation of resources for both plant metabolism and defence, as in-field fruits are connected to the plant, and secondary metabolites’ biosynthesis is not entirely based on the energetic resources present in detached plant tissues as it is at postharvest.

Although elicitors were initially used to increase plant tolerance to pathogens, the mechanism of induced resistance has been found to be involved in the activation of pathways of the plant’s secondary metabolism [8,9,10].

As secondary metabolites play an important role in the adaptation of plants to stress conditions, elicitors that simulate different biotic and abiotic stress conditions may trigger the plant biochemical system towards the increasing accumulation of secondary metabolites, including phenolic compounds [11,12,13]. For this reason, elicitors can be considered an interesting tool to obtain plants with a higher polyphenol content with positive consequences on the nutraceutical content of the fruit and vegetables [14].

Highbush blueberry (*Vaccinium corymbosum* L.) has gained popularity owing to its unique taste and its high content of bioactive compounds, such as phenolic compounds, with possible beneficial health properties. These health benefits, mainly due to the antioxidant activity, have been extensively described [15,16]. As a consequence, more interest has been placed on developing cultural management practices to enhance fruit polyphenolic levels and the exploitation of processing by-products as potential polyphenolic sources for the nutraceutical market. The possibility of further increasing the content of bioactive substances and, in parallel, improving the resistance of plants and fruits to adversity would further increase their nutritional and market value.

Among GRAS (generally recognized as safe) substances used as elicitors of plant defence response, chitosan is one of the most promising compounds studied. Chitosan is an inexpensive natural polysaccharide with high antimicrobial activity against a wide range of pathogenic and spoilage microorganisms, including fungi and bacteria. It shows a vast potential in many different areas, ranging from agriculture, as a possible elicitor of plant defence responses and as seed coating agent, to food systems as an additive, the cosmetic industry as a skin care and hair care ingredient and medicine as a food supplement with a hypocholesterolemic effect [17,18,19,20].

Many publications have reported the antifungal and elicitor effects of chitosan, ranging from in vitro studies [21,22,23] to applications in postharvest storage life and as a natural antifungal edible coating [3,24,25,26].

Numerous works have been conducted using treatments with chitosan in postharvest, but on the contrary, there are few reports in which this substance is used in preharvest. The effects of preharvest treatments with chitosan have been studied on perennial fruit trees like jujube [27], table grape [28], grapevine [29], apple [30], peach [31,32], sweet cherry [33], date palm [34] and apricot [35], as well as on horticultural crops such as tomato [36,37] and muskmelon [38].

Another elicitor that, in recent years, has attracted attention and received much interest is melatonin. Melatonin is an indolamine with amphiphilic characteristics acting as multifunctional signalling molecule. In plants, it is involved in the regulation of many different developmental processes regarding vegetative growth and reproductive behaviour [39,40]. This molecule promotes seed viability and germination, can stimulate root growth-effecting root apparatus architecture, can control floral transition and delay flowering and can improve fruit yield and crop quality by regulating fruit ripening. These effects are linked to its high antioxidant and free radical scavenging activity that protects tissues from oxidative stress and plays a pivotal role in defending plants against environmental stresses and in plant responses to harmful environmental factors [41,42] Melatonin also can enhance cellular antioxidant defence mechanisms by stimulating antioxidant enzymes and regenerating endogenous antioxidants [43]. It may also stimulate the biosynthesis of bioactive compounds and molecules that protect membrane structure against peroxidation [44,45,46].

To date, reports on preharvest melatonin treatments have investigated sweet cherry [47,48], date palm [49], apricot [50], pomegranate [51,52], japanese plum [53], Nanhong pear [46], eggplant [54] and tomato [55].

The aim of this study was to evaluate the effect of preharvest treatments with chitosan and melatonin on fruit quality traits and the nutritional parameters of three different blueberry cultivars. Particular interest was devoted to the accumulation of bioactive compounds and to the anthocyanin profile. To our knowledge, this is the first report on the effects of preharvest treatments with chitosan and with melatonin on blueberry plants and fruits.

## 2. Results

### 2.1. Environmental Factors

The analysis of thermal anomalies of 2021 and 2022 in comparison with the climatic normal from 1991 to 2020 highlights the peculiar features of the two seasons. Table 1 shows the monthly number of anomalies for daily minimum and maximum temperature. Based on the statistical distribution of historical data, each day is classified as characterized by no anomaly (NORM), mild positive anomaly (PAM), strong positive anomaly (PAS), mild negative anomaly (NAM) and strong negative anomaly (NAS). The 1991–2020 monthly average of each day is reported in brackets, showing the average distribution of anomaly along the twelve months compared to the norm. In this way, it is possible to understand when the month of a specific year is characterized by a peculiar behaviour differing from the average.

Overall, 2021 was an average year. January was warm, with particular reference to minimum temperature, while February and March showed average thermal conditions. The spring was mildly cold, as testified by the anomalies of April (on both minimum and maximum temperature) and May (with six mild negative anomalies of minimum temperature and the absence of positive anomalies of maximum temperature). The month of June was a particularly average month with 28 normal days of maximum temperature, while July had no cases of positive anomaly of maximum temperature. August and September were normal months, except for the minimum temperatures of September, characterized by a relevant number of mild positive anomalies. The final part of the year was characterized by very normal maximum temperatures, while in the case of minimum temperature October had a large number of mildly negative anomalies and November of mild positive ones.

The year 2022 was strongly characterized by warm conditions. More in detail, January was characterized by very normal conditions, February exhibited warm conditions and March was very variable. After mild cold conditions in April, warming characterized the following part of the year, with a decrease in the number of normal days and an increase in the number of mild and strong positive anomalies, especially during summer.

**Figure 1:**
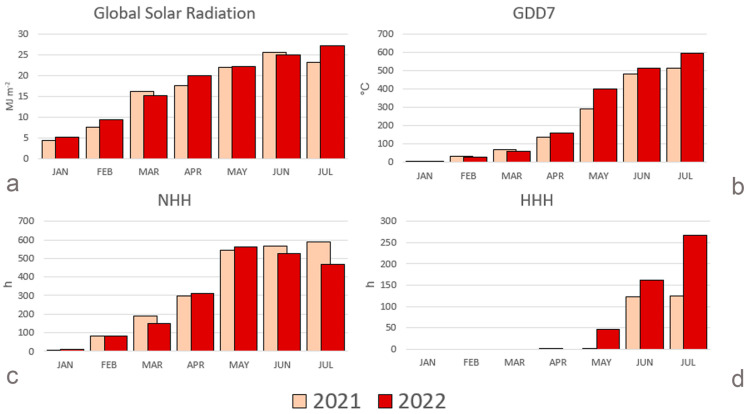
Monthly course of agrometeorological indices for 2021 and 2022. GSR = Global Solar Radiation [MJ m^−2^], GDD7 = accumulation of growing degree days with base temperature of 7 °C [°C], NHH = accumulation of hourly thermal resources [h], HHH = accumulation of hourly thermal excess [h].

The characterization of the environmental resources for blueberry was performed by considering global solar radiation (GSR), the accumulation of growing degree days with a base temperature of 7 °C (GDD7), the accumulation of hourly thermal resources (NHH) and hourly thermal excess (HHH, the latter two based on the normal heat hour approach [56] (Figure 1), focusing on the January–July period of 2021 and 2022. With reference to the availability of solar radiation (GSR), the period January–July 2022 was characterized by +6% of GSR when compared to 2021, with lower levels of radiation only in March (−7%) and June (−3%). The accumulation of GDD7 was higher in 2022 (+15%). The warming of the April–July period (+17%) drove this change. As explained in the Methods section, GDD7 does not consider the negative effects of high temperature. The normal heat hours accumulation shows a decrease in thermal resources that are useful for blueberry in 2022 (−7%), with a strong effect in June and July (−7% and −21%, respectively). This decrease is caused by thermal excess, as shown by the accumulation of HHH that characterizes the period of May–July (+90% in 2022).

### 2.2. Fruit Quality Parameters

#### 2.2.1. Dry Matter

**Figure 2:**
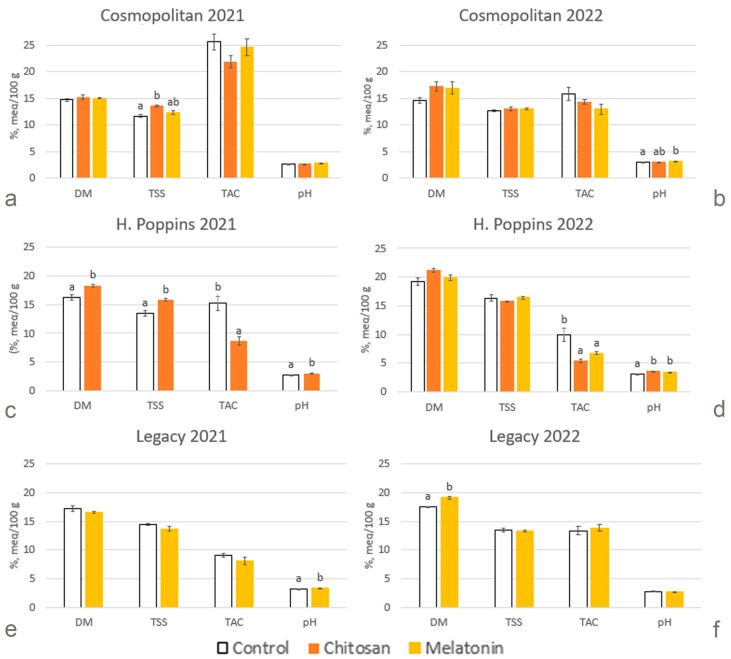
Effect of elicitor treatments on dry matter (DM, %), total soluble solids (TSS, %), total titratable acidity (TAC, meq/100 g FW, milliequivalents per 100 grams of fresh weight) and pH in cvs. ‘Cosmopolitan’, ‘H. Poppins’ and ‘Legacy’ in two subsequent harvest years (2021 and 2022). Values represent means ± SE (n = 4). Different letters indicate statistical differences (*p* ≤ 0.05) between treatments for each cultivar in each year.

Dry matter ranged from 14.6 to 16.2% in ‘Cosmopolitan’ (Figure 2a,b), from 17.8 to 19.9% in ‘Hortblue Poppins’ (‘H. Poppins’) (Figure 2c,d), and from 17.3 to 17.8% in ‘Legacy’ (Figure 2e,f). In 2022, the untreated fruit of ‘Cosmopolitan’ had significantly lower dry matter percentage than ‘Legacy’ and ‘H. Poppins’. Differences between subsequent years were recorded in ‘H. Poppins’ which increased dry matter by 18% and 15% in control and chitosan-treated berries, respectively, and in melatonin-treated fruit of ‘Legacy’ (+15%). The percentage was affected in 2021 in ‘H. Poppins’ by chitosan (+15%) and in 2022 in ‘Legacy’ by melatonin (+15%).

#### 2.2.2. Total Soluble Solids

As for dry matter, the content of total soluble solids was lowest in control berries of ‘Cosmopolitan’ in 2021 (Figure 2a) and significantly higher in those of ‘H. Poppins’ (Figure 2c) and of ‘Legacy’ (Figure 2e). An increase was recorded only in the untreated fruit of ‘H. Poppins’ in 2022 compared to 2021. An effect of treatments was only evident in 2021 with chitosan increasing the total soluble solids content in ‘Cosmopolitan’ (+16%) and ‘H. Poppins’ (+18%) (Figure 2a,c).

#### 2.2.3. Total Titratable Acidity

Considering control berries, ‘Cosmopolitan’ was the cv. which displayed the highest titratable acidity with an average of 20.7 meq 100 g FW^−1^ (Figure 2a,b). The lowest and significantly different level of acidity was showed by ‘Legacy’ in 2021 (Figure 2e) and by ‘H. Poppins’ in 2022 (Figure 2d) with 9.1 meq 100 g FW^−1^ and 9.9 meq 100 g FW^−1^, respectively. Still in untreated berries, there were significantly lower levels in titratable acidity across years in ‘Cosmopolitan’ and ‘H. Poppins’ (−38% and −35%, respectively) and an enhancement in ‘Legacy’ (+46%). A similar trend was also observed after treatments: in the second year of harvest, in ‘Cosmopolitan’, levels were reduced by 34% and by 47% in the berries treated with chitosan and melatonin, respectively, and levels were reduced in ‘H. Poppins’ by 37% in the berries treated with chitosan. On the other hand, melatonin-treated fruit of ‘Legacy’ showed a 72% increase in titratable acidity in 2022 compared to 2021. Elicitor treatments had no effect on ‘Cosmopolitan’ and ‘Legacy’, while ‘H. Poppins’ responded with a decrease in acidity in 2021 by 43% after chitosan treatment (Figure 2c) and in 2022 by 45% and 32% after chitosan and melatonin treatments, respectively (Figure 2d).

#### 2.2.4. pH

The pH trend is linked to that of acidity, with few differences (Figure 2). Considering untreated berries, the lowest pH was shown by ‘Cosmopolitan’ in 2021 (Figure 2a) and by ‘Legacy’ in 2022 (Figure 2f), although the latter was not significantly different from ‘Cosmopolitan’. Over the years, the trend is the same as shown by titratable acidity, with higher pH in 2022 in berries of ‘Cosmopolitan’ and ‘H. Poppins’ and lower pH in those of ‘Legacy’. In response to elicitor treatments, there was an increase in pH in all cvs.; in 2021, in treated an increase was observed in berries of ‘H. Poppins’ and ‘Legacy’ (Figure 2c,e), and in 2022, an increase was observed in ‘Cosmopolitan’ after melatonin treatment and in ‘H. Poppins’ after melatonin and chitosan treatments (Figure 2b,d).

#### 2.2.5. Total Phenolics

**Figure 3:**
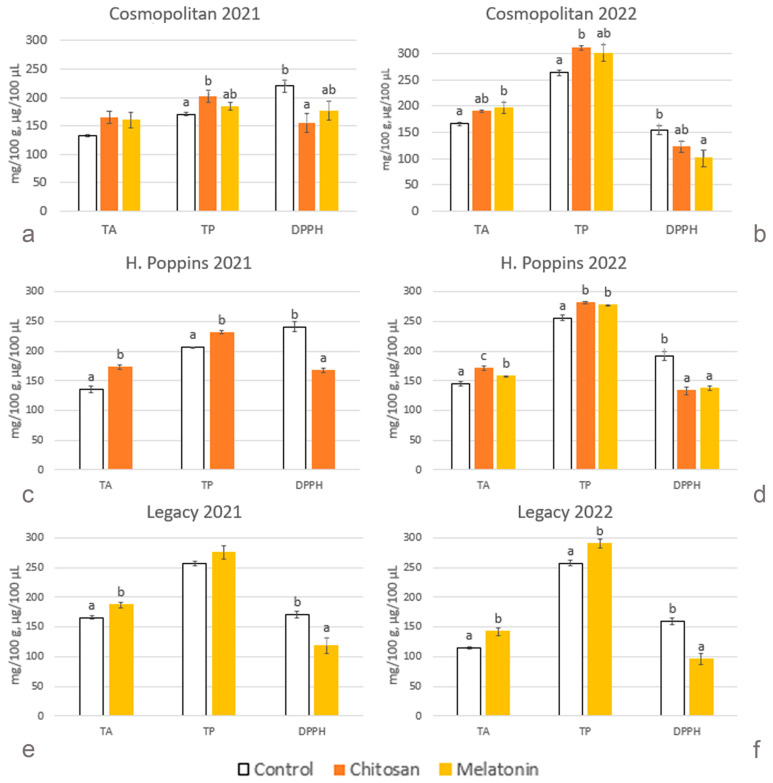
Effect of elicitor treatments on total anthocyanin content (AT, mg/100 g FW), total polyphenol content (TP, mg/100 g FW) and antioxidant activity (DPPH, IC 50%, µg/100 µL) in cvs. ‘Cosmopolitan’, ‘H. Poppins’ and ‘Legacy’ in two subsequent harvest years (2021 and 2022). Values represent means ± SE (n = 4). Different letters indicate statistical differences (*p* ≤ 0.05) between treatments for each cultivar in each year.

Control berries of the three cvs. were significantly different in phenols in 2021, i.e., content was the lowest in ‘Cosmopolitan’ (170 mg/100 g FW) (Figure 3a) and the highest in ‘Legacy’ (257 mg/100 g FW) (Figure 3e). In 2022, on the other hand, there were no differences among cvs. (Figure 3b,d,f). Consequently, untreated ‘Cosmopolitan’ and ‘H. Poppins’ accumulated more phenolic compounds in 2022 than in 2021 (+55 and +24%, respectively), while phenolic levels remained unchanged in ‘Legacy’. The same trends were present in the case of treated berries. Across years, in ‘Cosmopolitan’, phenolic levels increased by 49 and 64% in the fruits treated with chitosan and melatonin, respectively, and phenolic levels increased in ‘H. Poppins’ by 21% in the berries treated with chitosan. Generally, elicitor treatments increased the total phenolic content. Chitosan stimulated phenolic accumulation in both years in ‘Cosmopolitan’ (+19% in 2021 and +18% in 2022) and in ‘H. Poppins’ (+12% in 2021 and +10% in 2022), whereas in 2022, melatonin induced higher levels in ‘H. Poppins’ and in ‘Legacy’ (+9% and +13%, respectively). In ‘Cosmopolitan’ fruits treated with melatonin, in both years, the levels of total phenolics were intermediate between those of the control and the chitosan treatment and not significantly different. Considering total phenolics across years, all fruits of ‘Cosmopolitan’ and ‘H. Poppins’, untreated and treated, markedly increased in their phenolic contents, whereas no changes were observed in ‘Legacy’.

#### 2.2.6. Total Anthocyanins

Comparing total anthocyanins of the untreated cvs., in 2021, the fruits of ‘Legacy’ showed the highest content (166 mg/100 g FW) (Figure 3e), significantly greater than those of ‘Cosmopolitan’ and ‘H. Poppins’ (132 mg/100 g FW and 135 mg/100 g FW, respectively) (Figure 3a,c). Conversely, in 2022, ‘Cosmopolitan’ berries were the richest (166 mg/100 g FW) (Figure 3b) and ‘Legacy’ berries the poorest (114 mg/100 g FW) (Figure 3f). Consequently, comparing control fruits in the two years, an increase in anthocyanin content was found in ‘Cosmopolitan’, and a decrease was found in ‘Legacy’, while the levels remained stable in ‘H. Poppins’. The positive effects of elicitor treatments were evident in all the cvs. in both years except for ‘Cosmopolitan’ in the first sampling year. Chitosan positively affected the anthocyanin content in ‘H. Poppins’ by 28% in 2021 and by 18% in 2022, while in 2022, the ‘Cosmopolitan’ treated berries showed intermediate levels not significantly different from the control nor from the melatonin-treated fruits. Melatonin stimulated anthocyanin accumulation in ‘Legacy’ in both years, i.e., by 13% in 2021 and by 25% in 2022. In 2022, melatonin treatment resulted in anthocyanin levels significantly increasing by 18% in ‘Cosmopolitan’ and 9% in ‘H. Poppins’. Considering the differences in total anthocyanin levels between years, untreated ‘Cosmopolitan’ berries showed an increase in 2022 compared to 2021, while the treated ones did not. Anthocyanin levels of treated berries of ‘Legacy’ and ‘H. Poppins’, in contrast, mirrored those of control fruits, with a decrease and no change, respectively, between the two years.

#### 2.2.7. Antioxidant Activity

The antioxidant capacity evaluated by DPPH is expressed as IC50. Lower IC50 values in the DPPH radical scavenging activity determination are related to reductions in sample concentrations required to scavenge the DPPH radical by 50%, indicating an improvement in the antioxidant capacity of the fruit phenolic extract.

Considering the antioxidant activity in untreated berries, in 2021, fruit of ‘Legacy’ showed significantly higher activity (171 µg/100 µL) (Figure 3e) than those of ‘Cosmopolitan’ (220 µg/100 µL) (Figure 3a) and ‘H. Poppins’ (241 µg/100 µL) (Figure 3c). On the other hand, in 2022, both ‘Cosmopolitan’ and ‘Legacy’ showed stronger antioxidant capacity (155 µg/100 µL and 160 µg/100 µL, respectively) (Figure 3b,f) than ‘H. Poppins’ (192 µg/100 µL) (Figure 3d).

Comparing the values across years, in 2022, the antioxidant capacities of untreated ‘Cosmopolitan’ and ‘H. Poppins’ were significantly higher (+29% and +20%) than in 2021, while that of untreated ‘Legacy’ did not change.

Elicitor treatments markedly enhanced the antioxidant activity in ‘H. Poppins’ (+30% in 2021 after chitosan treatment and +31% and +28% after chitosan and melatonin treatments, respectively, in 2022) and ‘Legacy’ (+31% in 2021 and +40% in 2022), while in ‘Cosmopolitan’, significant differences were reached by chitosan in 2021 (+30%) and by melatonin in 2022 (+35%).

#### 2.2.8. Anthocyanin Profile

The total amounts of anthocyanins, calculated as the sum of the individual anthocyanins measured by the chromatographic profile, are consistent with the results obtained by the spectrophotometrical analysis. The identified anthocyanins were monogalactosides (gal), monoarabinosides (ara) and monoglucosides (glu) of delphinidin (Dp), cyanidin (Cy), petunidin (Pt), peonidin (Pn) and malvidin (Mv). In ‘Cosmopolitan’, the acetylated forms of Dp, Cy, Pt, Pn and Mv were detected, but these were lacking in ‘H. Poppins’ and ‘Legacy’ (Supplementary Tables S1 and S2). To better understand the variations in the anthocyanin composition, the identified anthocyanin compounds were grouped on the basis of the anthocyanidin present in the molecular structure. In all cvs., the predominant anthocyanidins were the tri-hydroxylated (in the B ring) Mv followed by Dp and Pt. The di-hydroxylated Cy and Pn were present in minor absolute and relative amounts.

**Figure 4:**
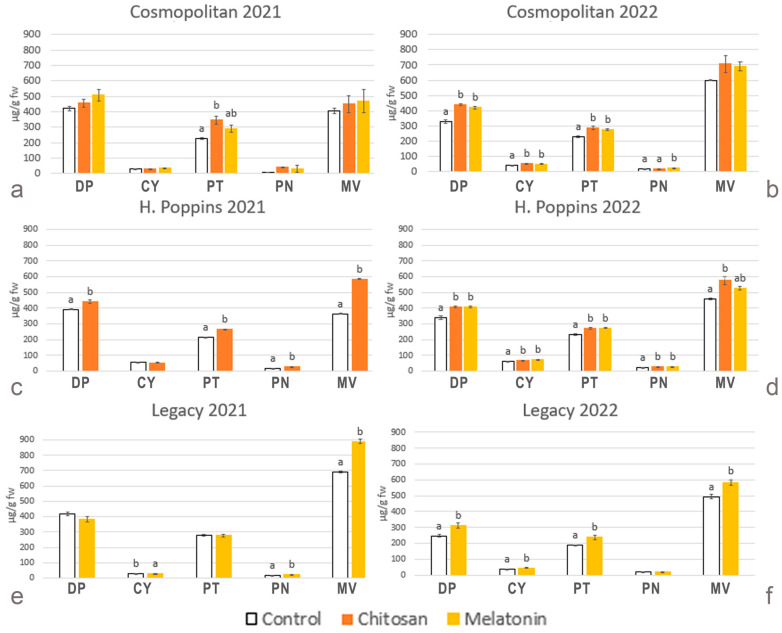
Effect of elicitor treatments on total amounts of individual anthocyanins (clustered into the representative anthocyanidin classes) in cvs. ‘Cosmopolitan’, ‘H. Poppins’ and ‘Legacy’ in two subsequent harvest years (2021 and 2022). Values represent means ± SE (n = 4). Different letters indicate statistical differences (*p* ≤ 0.05) between treatments for each cultivar in each year.

Considering the anthocyanins of untreated berries, the highest amounts of Mv and Dp were detected in 2021 in ‘Legacy’ (693 µg/g FW) (Figure 4e) and ‘Cosmopolitan’ (420 µg/g FW) (Figure 4a), respectively, and in 2022, the highest amounts were detected in ‘Cosmopolitan’ (600 µg/g FW) (Figure 4b) and in ‘H. Poppins’ (341 µg/g FW) (Figure 4d). In 2021, Pt reached the highest levels in ‘Legacy’ (277 µg/g FW) (Figure 4e), and in 2022, Pt reached the highest levels in ‘H. Poppins’ (233 µg/g FW) (Figure 4d). In both years, the cv. richest in di-hydroxylated Cy and Pn was ‘H. Poppins’, with 55 and 17 µg/g FW, respectively, in 2021 (Figure 4c) and 60 and 22 µg/g FW in 2022 (Figure 4d). After elicitor treatments, the plant responses were different depending on the cv. and the harvest year. In ‘Cosmopolitan’, in 2021, only the content of Pt in chitosan-treated berries increased, while in 2022, the levels of Dp, Pt and Cy were enhanced by chitosan and melatonin and those of Pn only by melatonin. ‘H. Poppins’ responded to elicitors by increasing the content of all different classes of anthocyanidins in both years, except Cy in 2021. In ‘Legacy’, melatonin stimulated the accumulation of Mv, Cy and Pn in 2021, whereas in 2022, all types of anthocyanidins increased except Pn (Figure 4e,f).

**Figure 5:**
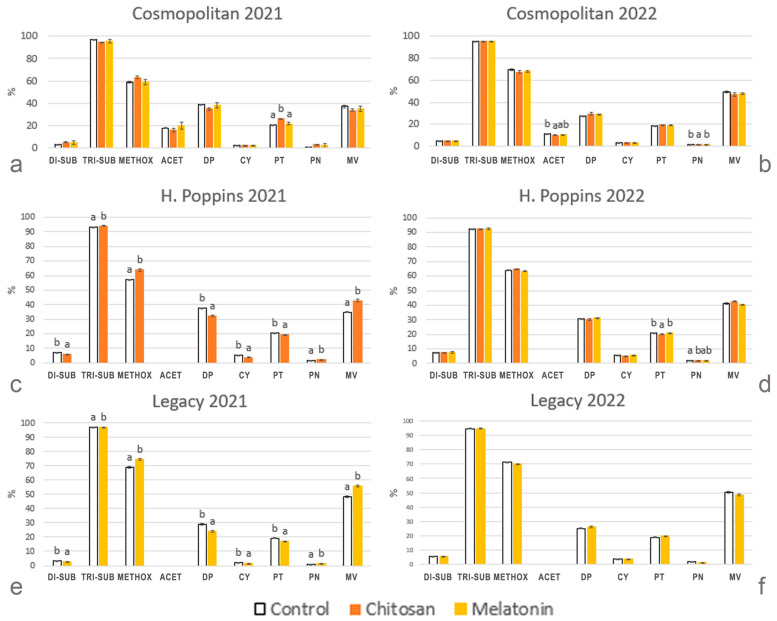
Effect of elicitor treatments on relative proportion of individual anthocyanins (clustered into the representative anthocyanidin classes) and of di-hydroxylated (DI-SUB), tri-hydroxylated (TRI-SUB), methoxylated (METHOX) and acetylated (ACET) forms in cv. ‘Cosmopolitan’, ‘H. Poppins’ and ‘Legacy’ in two subsequent harvest years (2021 and 2022). Values represent means ± SE (n = 4). Different letters indicate statistical differences (*p* ≤ 0.05) between treatments for each cultivar in each year.

In addition to the absolute contents, it is useful to express anthocyanin composition in relative terms. Considering the relative proportion of the single anthocyanins, the effects of the treatments with elicitors depended on the cv. and on the harvest year, similar to the case for the absolute concentrations but in different terms. In ‘Cosmopolitan’, in 2021, chitosan increased the percentage of Pt (Figure 5a), and in 2022, chitosan decreased that of the acetylated forms and of Pn (Figure 5b). In chitosan-treated ‘H. Poppins’ berries, all relative proportions of anthocyanidins were affected in 2021, with increases in tri-hydroxylated forms (sum of Mv, Dp, Pt), in methoxylated forms (sum of Mv, Pt, Pn) and in Mv and decreases in the di-hydroxylated forms (sum of Cy, Pn), Dp, Cy and Pt (Figure 5c). In 2022, however, chitosan only affected Pt with a decrease and Pn with an increase (Figure 5d). In 2021, the effect of melatonin on ‘Legacy’ consisted of enhanced proportions of the tri-hydroxylated and methoxylated forms, Pn and Mv and decreased proportions of di-hydroxylated anthocyanidins, Dp, Cy and Pt (Figure 5e). In contrast, in 2022, no variation was recorded for the treated berries (Figure 5f).

## 3. Discussion

Cropping systems are evolving towards agronomic strategies that can reduce the environmental impact and improve the sustainability of their production. Elicitors can act as priming stimuli to which a plant responds by acquiring a memory that boosts induced defences and resistance against a broad range of pathogens. This acquired protection could reduce the need for synthetic fungicides. Chemical elicitors such as chitosan and melatonin are non-toxic natural compounds and represent an attractive option for sustainable and organic agriculture, minimizing the impact on human health and the environment and contributing to the prevention of the development of pathogen resistance to certain fungicides, while covering the demand for residue-free plant products. Moreover, these substances can generally induce low-cost metabolic changes in the plant, including the synthesis of different classes of secondary metabolites such as phenolic compounds that contribute to the enhancement of antioxidant activity and nutritional quality, which leads to a longer storage potential and shelf life [14]. In relation to diet and human health, polyphenols are antioxidant bioactive compounds that are important in the prevention of chronic diseases such as cardiovascular disorders, cancers, diabetes and neurodegenerative diseases [57]. Therefore, elicitor treatments may represent a sustainable and inexpensive strategy to increase the nutraceutical quality of fruits. In our study, chitosan and melatonin proved to be effective in stimulating different blueberry plant responses. Regarding the effect of elicitor treatments on the quality parameters related to the ripening stage, ‘Cosmopolitan’ generally did not change these parameters and only total soluble solids were higher in 2021, while pH slightly decreased in 2022. On the other hand, ‘H. Poppins’ reacted more extensively to chitosan treatment. In 2021, all parameters were affected, i.e., dry matter and total soluble solids increased, while titratable acids diminished, altering pH, and in 2022, chitosan similarly affected titratable acidity by lowering it and pH by raising it. These data agree with previous studies that reported slight effects of chitosan total soluble solids and titratable acidity in blackberry [58]. The preharvest treatments of this study were conducted with a water-soluble chitosan. On the contrary, in many postharvest research studies, fruits were dipped in an acidic solution of a practical-grade chitosan, which forms a semi-permeable film on the surface of the fruit, decreasing gaseous exchange with a positive effect on titratable acidity and giving a glossy appearance to the fruit. In blueberries, a postharvest chitosan coating delayed ripening as indicated by lower respiration rate, higher total soluble solids content and titratable acidity [59]. Also, when used in preharvest, this formulation showed viscous properties that affect the internal gas composition of fruit by producing a film coating on the surface and maintaining a higher titratable acidity, as reported by Gayed et al. on peaches [32]. This property of chitosan as a film-forming solution and edible coating is absent in treatments with water-soluble chitosan, which, however, is more practical when sprayed and does not alter the appearance of the fruit. As for chitosan, melatonin did not exert in ‘Cosmopolitan’ any effect on ripening parameters in both years. In the same way, in 2021 and 2022, melatonin-treated ‘Legacy’ berries increased only the pH and the dry matter, respectively. On the other hand, in 2022, the response of ‘H. Poppins’ to melatonin was the same as shown after chitosan treatment, linked to a decrease in titratable acidity accompanied by a pH raise.

Differently from quality/ripening parameters, elicitor treatments markedly affected the levels of phenylpropanoid and antioxidant compounds. In particular, total anthocyanins and total phenolics accumulation were stimulated in all cvs. in both years. Considering chitosan treatment, in ‘Cosmopolitan’, chitosan increased total phenolics and influenced total anthocyanin content and antioxidant capacity positively, although not significantly. In ‘H. Poppins’, the effect of chitosan was more pronounced, and all the parameters linked to nutraceutical content, i.e., total phenolic and anthocyanin contents and antioxidant capacity, were enhanced. Melatonin caused similar responses to chitosan treatment in ‘Cosmopolitan’ in both years and in ‘H. Poppins’ in 2022, enhancing phenolic and anthocyanin accumulation and increasing antioxidant potential. The melatonin-treated fruit of ‘Legacy’ also showed a significant improvement in nutraceutical and antioxidant properties.

Comparing the two harvesting years, the behaviour of ‘Cosmopolitan’ and ‘H. Poppins’ was similar. In 2022, the berries of both cvs. showed, compared to 2021, a significant increase in total phenolics, accompanied by an enhancement in antioxidant capacity and a considerable reduction in titratable acidity linked to a raise in pH. On the contrary, in ‘Legacy’ in 2022, the levels of phenolic compounds and antioxidant power remained unchanged compared to 2021, but the amounts of total anthocyanins declined. Likewise, Feliziani et al. reported differences in the effectiveness of preharvest treatments with inducers in controlling postharvest decay between two strawberry varieties due to possible activation of cultivar-specific pathways [33]. Different responses to a specific elicitor treatment were also observed among six clones of Monastrell grape, suggesting the need to conduct preliminary exploratory field research to evaluate the possible variation in responses between clones from the same variety and between seasons, since clone–environment interactions may also exist [60]. Moreover, genetic background affected the responses to other elicitors such as benzothiadiazole in mango [61] and jasmonic acid seed treatment in tomato [62].

Regarding the impact of elicitor treatments on the anthocyanin profile, a positive effect is shown for total anthocyanin accumulation. The effects of chitosan and melatonin were quite comparable and, generally, the amounts of all anthocyanin classes improved in both years, largely in 2022. In addition to absolute concentrations, it is useful to evaluate the composition of anthocyanin in relative terms. Considering the relative proportion of individual anthocyanins, responses to elicitor treatments were different among cvs. and even more markedly different between years. ‘Cosmopolitan’ generally did not change in anthocyanin proportions after treatments in both years, while in 2021, an enhancement in the proportion of tri-substituted and methoxylated molecular structures was observed in ‘H. Poppins’ and ‘Legacy’, mainly caused by a steep increase in Mv that partially occurred at the expenses of Dp and Pt. Moreover, Cy decreased, while Pn slightly increased. On the contrary, in 2022, both ‘H. Poppins’ and ‘Legacy’ did not change the anthocyanin profile in response to treatments. In particular, treatments did not cause the marked increase in Mv seen in 2021 and, consequently, no significant decreases in the proportions of the other tri-substituted Dp and Pt were observed.

These contradictory data obtained in the two growing years are mainly attributable to the different climatic conditions of the two harvest years. In 2022, the higher temperatures experienced by the plants during the last ripening period determined a heat stress. Metabolic processes such as respiration and photosynthesis are very sensitive to temperature increases. Heat stress, which is often accompanied by drought, may significantly decrease stomatal conductance and cause higher respiration rates at the expense of organic acids, the main substrates of respiration in blueberry fruit [63]. In fact, the higher temperatures recorded in 2022 compared to 2021 resulted in a significant reduction in the acidity of the fruit of ‘Cosmopolitan’ and ‘H. Poppins’, consequently increasing the pH value. Similar effects are reported in several studies on grape [64,65]. Conversely, these cvs. showed no changes in anthocyanin content and higher phenolic levels in 2022, unlike grapes, which generally respond to high temperatures (over 30 °C) by lowering anthocyanin content [63]. ‘Legacy’ showed the opposite behaviour with respect to ‘Cosmopolitan’ and ‘H. Poppins’ and showed higher acidity and lower anthocyanin content in 2022 compared to 2021. In ‘Legacy’, heat stress may not affect respiration but rather anthocyanin metabolism.

Blueberries are typically classified into different groups depending on their chilling requirements for the onset of flowering. Highbush blueberry varieties with high chilling requirements, typically grown in cold winter regions, are named northern highbush. Instead, cvs. with low chilling requirements (less than about 550 chilling hours, <7 °C) are named southern highbush and are interspecific hybrids derived from crosses between the northern highbush and *Vaccinium* species both native and adapted to the southeastern United States with lower chilling requirements, such as *V. darrowii* Camp. ‘Cosmopolitan’ and ‘H. Poppins’ are northern highbush blueberries, whereas ‘Legacy’ belongs to the southern group [66].

These different genetic backgrounds affected both heat stress responses and elicitor treatment effects. A marked difference concerns the acidity of the fruit in the two different years. Decreases in organic acid content due to high temperatures were likely associated in the two northern highbush blueberries with higher respiration rates, in contrast to the southern highbush ‘Legacy’. Modifications in organic acid levels may be associated with different respiration rates but also with different plant resource allocations. In fact, in 2022, characterized by higher summer temperatures and heat stress compared to 2021, ‘Cosmopolitan’ and ‘H. Poppins’ had a higher total phenolic accumulation and total anthocyanin content remained unchanged, while ‘Legacy’ showed no changes in levels of phenols and allocated fewer resources to the accumulation of anthocyanins. However, a different response to elicitor treatment was shown by ‘H. Poppins’ and ‘Legacy’, which could imply a different allocation of resources. The response to treatment in ‘H. Poppins’ is a marked increase in the content of anthocyanins and total phenolics accompanied by a decrease in acidity, while in ‘Legacy’, the increase in the phenolic compounds is not associated with a variation in acidity.

In berries, apart from total anthocyanin concentrations, changes in the contents and relative proportions of individual anthocyanins were related to high temperature stress. In ‘Cosmopolitan’ and in ‘H. Poppins’, compositional changes related to heat stress were associated with the increased proportions of Mv and reduced proportions of Dp, as reported also by many authors in grapevine [64,65,67]. Moreover, in accordance with these changes, Timmers et al. [68] observed that across highbush blueberry genotypes, the percentages of Dp and Mv glycosides mirrored each other in the relative proportion during the harvest season, with an increasing trend in Mv percentage during almost the entire harvest period and a parallel decrease in Dp. These two forms of anthocyanins are related because they are found within the same section of the anthocyanin biosynthetic pathway. In fact, Dp glycosides undergo two-step methylation to form Mv glycosides. The intermediate in this two-step methylation is Pt, which decreased in relative content in 2022 compared to 2021 in ‘Cosmopolitan’ but not in ‘H. Poppins’.

In the warmer year of 2022, ‘Legacy’ showed an increase in the relative levels of the methoxylated forms and also, but not reaching significance, in Mv and a decrease in Dp proportions in untreated berries. On the other hand, it showed a decrease in relative levels of the methoxylated forms and Mv and a parallel increase in the proportions of Dp in treated fruit. ‘Cosmopolitan’ and ‘H. Poppins’ showed also increased proportions of methoxylated pigments in 2022 regardless of treatment. Berries of ‘Cosmopolitan’ accumulated acetylated anthocyanins in higher proportions under heat stress conditions. A similar response to high summer temperatures was reported in grape. Three grape varieties under a two-crop-a-year cultivation system accumulated significantly higher levels of methoxylated anthocyanins, and one variety also accumulated acetylated anthocyanins in summer compared to winter [69]. A progressive increase in the proportion of the more stable tri-substituted and methoxylated pigments occurs also during ripening [70].

Considering the anthocyanin content in absolute terms, in ‘Cosmopolitan’ and ‘H. Poppins’, the changes across years in Mv and Dp reflected those shown in relative terms and are in line with that published by Venios et al. [64]. Moreover, in all the fruits of the two cvs., the accumulation of Cy was higher under conditions of greater thermal stress, i.e., in 2022, as reported in Kyoho grape by Cheng et al. [71].

The antioxidant capacity mirrored the contents of anthocyanins and phenolic compounds and was always higher in treated berries, regardless of cv. and harvest year, and it is linked to enhanced fruit quality. The positive effect of elicitor treatments on the accumulation of nutraceutical antioxidant compounds did not lead to significantly different IC50 values only in the case of ‘Cosmopolitan’ in 2021, although the trend is similar to the other responses to treatment. Moreover, similar beneficial effects on phenolic content and antioxidant activity were obtained with melatonin preharvest application on Merlot grape [45], sweet cherry [72] and Elvira blackberry [73]. The stimulation of phenol and anthocyanin accumulation and an increase in antioxidant activity after preharvest treatment with chitosan was also reported by He et al. on strawberry [74], by Lo Piccolo (2023) on raspberries [75] and by Griñán et al. on pomegranate [76]. Antioxidant phytochemicals play an important role in the prevention and treatment of chronic diseases, and the consumption of fruits with high antioxidant capacity is, therefore, beneficial [77].

## 4. Materials and Methods

### 4.1. Chemicals

Standards of cyanidin (Cy)-, delphinidin (Dp)-, petunidin (Pt)-, peonidin (Pn)-, malvidin (Mv)- and their 3-O-glucoside (glc), Cy-, Pn-, Pt-, Mv-3-O-galactoside (gal) and Cy-arabinoside (Cy-ara) were purchased from Polyphenols Laboratory (Sandnes, Norway). Methanol, acetonitrile, phosphoric, trifluoroacetic acid (TFA) and melatonin were from Merck (Darmstadt, Germany). Tween was from Sigma-Aldrich (Saint Louis, MO, USA), and chitosan was provided by SKL Fertilizzanti (Chieti, Italy). Soil for acidophilic plants was from Vegetal Radic Acid (Tercomposti S.P.A., Calvisano, Italy). Water was from Milli-Q apparatus (Millipore, Milford, MA, USA).

### 4.2. Plant Material and Treatments

Blueberry plants (*Vaccinium corymbosum* L.) were grown in Arcagna (45.33992° N; 9.45209° E) in Northern Lombardy (Italy) at 83 m above sea level. Seven-year-old plants of three different cultivars, ‘Cosmopolitan’, ‘Hortblue Poppins’ (‘H. Poppins’) and ‘Legacy’ were grown in 70 L pots in a soil for acidophilic plants, optimal for blueberry growth. Conventional farming practices and micro-irrigation were carried out in the field. Berry samples were collected 24 h after elicitor spray treatments with 1% chitosan or 100 µM melatonin + Tween 0.01%. Treatments were performed every two weeks starting from the appearance of fruit pigmentation throughout the growing season (in a total of 3 applications, with each application performed until dripping). Fruits from plants treated with water were also collected as a control. ‘Cosmopolitan’, an early ripening variety, is harvested in Northern Italy in mid-to-late June, whereas ‘H. Poppins’ and ‘Legacy’, mid-season varieties, ripen between the end of June and the first week of July. In 2021, fully pigmented ripe berries of ‘Cosmopolitan’ were collected on June 11th, of ‘H. Poppins’ on 21 June and of ‘Legacy’ on 2 July. In 2022, ‘Cosmopolitan’ and ‘H. Poppins’ were sampled on 27 June and ‘Legacy’ on 30 June. After elicitor application, four treated plants and four control plants were randomly selected for sampling. Plants were uniform in vegetative development and size. Consequently, the pool of berries harvested from the same plant represented one biological replicate for each cultivar, i.e., each sample subjected to chemical analysis was taken from the fruit pool of a single plant. Of each cv., the four treated and the four untreated plants provided the four control and the four treatment samples. In 2021, chitosan was applied to ‘Cosmopolitan’ and ‘H. Poppins’ and melatonin to ‘Legacy’. In 2022, the treatments were replicated as in 2021 and a treatment with melatonin to ‘H. Poppins’ was added to the experimental plan.

The total number of samples was 60 (in 2021, 4 individual plants treated or not with chitosan for ‘H. Poppins’, 4 individual plants treated or not with melatonin for ‘Legacy’ and 4 individual plants treated or not with chitosan or melatonin for ‘Cosmopolitan’; in 2022, 4 individual plants treated or not with chitosan or melatonin for ‘Cosmopolitan’ and ‘H. Poppins’ and 4 individual plants treated or not with melatonin for ‘Legacy’). Harvested fruits were then weighed, placed in plastic bags and stored at −80 °C until laboratory analysis.

### 4.3. Thermal and Radiative Analysis

The characterization of the environmental conditions across the two experimental seasons was based on the analysis of meteorological data collected by the weather station of Sant’Angelo Lodigiano, belonging to the ARPA Lombardia meteorological network [78]. The station is 8 km from the experimental site and provides a long time series of data, allowing a robust analysis of seasonal anomalies. The agreement between Sant’Angelo Lodigiano and the experimental farm data was previously verified with specific monitoring campaigns and subsequent comparison activities. Seasons 2021 and 2022 were characterized based on the climatic normal of 1991–2020. The characterization focuses on radiative and thermal analysis, since the drip irrigation system provided the optimal water supply during the two experimental seasons.

The thermal course of the two seasons was described by means of an anomaly classification for maximum and minimum daily temperature. The temperature (T) of each day of the year was classified based on the climatic normal statistics as follows:

1. NAS—Strong negative anomaly: T < AVG − 2SD
2. NAM—Mild negative anomaly: AVG − 2SD ≤ T < AVG − 1SD
3. NORM—No anomaly: AVG − 2SD ≤ T < AVG − 1SD
4. PAM—Mild positive anomaly: AVG − 1SD ≤ T < AVG + 1SD
5. PAS—Strong positive anomaly: T ≥ AVG + 2SD

where AVG and SD are the average and the standard deviation of the dataset of temperature for each day of the year in the 1991–2020 period.

The evaluation of the environmental resources and limitations for blueberry across the two seasons was based on the calculation of the following indices:

- GSR—Summation of daily global solar radiation [MJ m^−2^]
- GDD7—Accumulation of growing degree days with base temperature 7 °C [79] [GDD]. GDD represents the thermal resources that are useful for the development of blueberry. However, the absence of upper thresholds leads this index to the overestimation of the positive effect of high temperatures.
- NHH—Accumulation of normal heat hours [56,80], with a cardinal minimum of 7 °C [79], an optimal range between 20 °C and 26 °C [81] and a cardinal maximum of 32 °C [82] [NHH]. This approach weighs the hourly temperature by considering under-optimal, optimal and over-optimal temperatures.
- HHH—Accumulation of thermal excess [56,80] based on the same parameterization of NHH [HHH]. This is related to the previous index and accounts for the conditions of thermal excess.

### 4.4. Fruit Quality Parameters

#### 4.4.1. Dry Matter Content

To measure the dry matter content of the samples, six intact berries for each sample were freeze dried and weighed before and after the procedure until no more decrease in weight was recorded.

#### 4.4.2. Total Soluble Solids

Total soluble solids, expressed as °Brix, were determined by a hand refractometer (Atago mod., N1, Tokyo, Japan) on the juice obtained from squeezing 5 g of berries.

#### 4.4.3. Total Titratable Acidity and pH

Titratable acidity, expressed as meq 100 g FW^−1^, was measured by titrating 100 mL samples of 10% extracted juice with 0.1 M NaOH to pH 8.2 with an automatic sample titrator (TitroMatic 2S-3B, Crison Instruments, Barcelona, Spain).

#### 4.4.4. Total Phenolics and Total Anthocyanins Analysis

Approximately 10 g of frozen berries of equal size were ground in 20 mL of cold extraction solution containing EtOH/HCOOH/H_2_O (25/2/73). The homogenate was placed in an ultrasonic bath for 15 min. The suspension was centrifuged at 10,000× *g* for 20 min at 4 °C, and the supernatant was recovered. The pellet was resuspended with 10 mL of extraction solution and treated as described above. Then, the supernatants were combined, and the volume was adjusted to 40 mL by extraction solution. All extracts were stored at −80 °C before spectrophotometric analysis.

Total phenolic contents were determined following the Folin–Ciocalteau method. Briefly, 0.5 mL of Folin–Ciocalteau reagent, 4.45 mL of distilled water and 2 mL of 10% Na_2_CO_3_ were added to 0.05 mL of extract. The solution was immediately diluted to a final volume of 10 mL with distilled water. The optical density, after 90 min, was measured at 700 nm on a 10S UV-vis spectrophotometer (Thermo-Scientific, Waltham, MA, USA). Results were expressed as milligrams of gallic acid per 100 g FW. For all evaluations, duplicate reactions per replicate were carried out. Total anthocyanins were estimated on a 10S UV-vis spectrophotometer (Thermo-Scientific, Waltham, MA, USA) by measuring the absorption peak of the anthocyanin pigments at 530 nm and expressed as cyanidin 3-glucoside using a molar extinction coefficient of 26.900 L mol^−1^ cm^−1^ and reported in milligrams per 100 g FW.

#### 4.4.5. Antioxidant Activity

The free radical scavenging capacity of blueberry extracts was evaluated by the DPPH method. Four different dilutions of each extract obtained for total phenolic and anthocyanin analysis were tested, adding 430 μL of a DPPH solution (296 μM) to each sample diluted in ethanol [83]. The absorbances of the solutions were measured at 515 nm after incubation in the dark at 25 °C for 60 min. The scavenging rate was calculated as IC 50% for each of the extracts with the following formula:

$$\mathrm{IC}\%=\frac{\mathrm{Asample}-\mathrm{Ablank}}{\mathrm{Acontrol}-\mathrm{Ablank}}\;\times \;100$$

Asample: absorbance of the extract;

Acontrol: absorbance of the control reaction (reagents except the extract);

Ablank: ethanol absorbance.

#### 4.4.6. Anthocyanin Determination by UHPLC-DAD-HR-MS

The extract obtained for the analysis of total anthocyanins was also used, after dilution, for the qualitative and quantitative determination of individual anthocyanins. The chromatographic analysis was performed by a UHPLC Vanquish Flex (Thermo) coupled to a Vanquish HL PDA (Thermo) and an HR-MS Orbitrap mod. Q-Exactive model Focus (Thermo) equipped with an HESI-II probe for ESI. The separation was carried out using a 2.6 μm Kinetex C_18_ column (150 mm × 4.6 mm, Phenomenex, Torrence, CA, USA) protected with a guard column at 1.7 mL/min and a flow rate split 5:1 before exposure to an electrospray ionization (ESI) source. The column and sample were maintained at 45 and 20 °C, respectively. The eluents were (A) 0.2% TFA in water and (B) acetonitrile 0.2% TFA in water (35:65, *v*/*v*). The linear gradient was 0–15 min 14% B; 15–25 min from 14% to 20% B; 25–35 min from 20 to 32% B; 35–45 min from 32 to 50% B; 45–48 min 50 to 90% B; and 90% for 3 min. The HR-MS operative conditions were as follows: spray voltage +4.0 kV, sheath gas flow rate 60 (arbitrary units), auxiliary gas flow rate 20 (arbitrary units), capillary temperature 350 °C, capillary voltage +30 V, S-lens +80 V, and heater temperature 130 °C. The MS data were processed using Xcalibur 4.1 Software (Thermo Scientific, Rodano, MI, Italy). Mass spectrometry was performed through the infusion of a 5 μg/mL cyanidin-3-O-glucoside solution. The calibration curve in the range of 0.2–20 μg/mL was used for ACN quantification. Peaks were identified by evaluating accurate mass, fragments obtained in the collision cell, and the online UV spectra (220–650 nm).

### 4.5. Statistical Analysis

All data were subjected to ANOVA performed in IBM SPSS Statistics software, version 25 (SPSS Inc., Chicago, IL, USA), using general linear model univariate analysis with treatments, harvesting year, or cultivar as fixed factors. Significant differences among means were calculated by Tukey’s post hoc test. Differences at *p* ≤ 0.05 were considered as significant. Additional information is reported in the figure legends.

## 5. Conclusions

The results of the present study indicate that the preharvest spraying of melatonin and chitosan had a beneficial impact on improving the quality of blueberry in terms of nutraceutical content related to the content of anthocyanin and phenols and in terms of the antioxidant activity of fruit. The effect of elicitor treatments on other quality attributes, i.e., titratable acidity and anthocyanin composition, also depended on climatic factors and on genetic background, as diverse cvs. responded variously in harvest years characterized by different climatic conditions. The consumption of berries with increased polyphenol content and antioxidant activity has a beneficial effect on human health, with a positive impact on the prevention of different chronic diseases. To our knowledge, this is the first report showing the impact of preharvest treatments with melatonin and chitosan on blueberry fruit quality at harvest. The objective in using these elicitors is to minimize the impact on human health and the environment of using conventional agrochemicals to prevent fruit spoilage by fungal pathogens, obtaining residue-free fruits and, as shown in this research, improving quality attributes. This sustainable strategy can be successfully adopted, keeping in mind that some differences in the response to treatments are linked to the different genetic backgrounds of the blueberry varieties in relation also to the different climatic parameters to which they are subjected.

## Figures and Tables

**Table 1:** Monthly count of daily thermal anomalies in 2021 and 2022. Based on the statistical distribution of historical data, each day is classified as characterized by no anomaly (NORM), mild positive anomaly (PAM), strong positive anomaly (PAS), mild negative anomaly (NAM) or strong negative anomaly (NAS). The 1991–2020 monthly average of anomaly is reported in brackets.

2021										
Month	Minimum Daily Temperature					Maximum Daily Temperature				
	Nas	Nam	Norm	Pam	Pas	Nas	Nam	Norm	Pam	Pas
January	0 (0.7)	2 (4.0)	22 (21.7)	7 (3.9)	0 (0.7)	0 (0.2)	2 (5)	25 (21.1)	4 (3.9)	0 (0.8)
February	0 (1.0)	2 (3.1)	8 (19.5)	13 (4.0)	5 (0.4)	0 (0.7)	1 (3.9)	22 (19.1)	2 (3.8)	3 (0.5)
March	1 (0.6)	6 (4.3)	19 (20.9)	5 (4.9)	0 (0.3)	0 (0.9)	4 (4.1)	22 (21.1)	5 (4.5)	0 (0.4)
April	2 (1.2)	6 (3.6)	19 (20.6)	3 (4.3)	0 (0.3)	0 (0.4)	10 (4.9)	17 (19.6)	1 (4.8)	2 (0.3)
May	0 (0.8)	6 (4.1)	25 (21.2)	0 (4.8)	0 (0.1)	1 (0.9)	3 (4.2)	27 (21.3)	0 (4.3)	0 (0.3)
June	0 (0.7)	0 (3.8)	21 (20.9)	8 (4.2)	1 (0.4)	0 (0.9)	0 (3.8)	28 (20.4)	2 (4.7)	0 (0.2)
July	0 (0.9)	2 (4.4)	26 (20.9)	3 (4.5)	0 (0.3)	1 (1.1)	4 (3.7)	26 (21.8)	0 (4.1)	0 (0.3)
August	0 (0.8)	6 (4.3)	22 (20.7)	1 (5.0)	2 (0.2)	2 (0.9)	4 (3.6)	21 (22.3)	3 (3.7)	1 (0.5)
September	0 (0.8)	0 (4.3)	21 (20.0)	8 (4.8)	1 (0.1)	0 (0.8)	1 (3.9)	26 (20.4)	3 (4.7)	0 (0.3)
October	0 (0.7)	10 (4.1)	16 (21.4)	3 (4.6)	2 (0.2)	0 (0.7)	3 (4.1)	27 (21.3)	1 (4.3)	0 (0.6)
November	0 (0.4)	5 (4.8)	16 (19.4)	9 (5.0)	0 (0.4)	0 (0.7)	4 (3.8)	23 (21)	3 (3.9)	0 (0.6)
December	0 (0.4)	3 (4.4)	23 (21.2)	4 (4.1)	1 (0.9)	0 (0.7)	7 (4.1)	23 (21.2)	1 (4.4)	0 (0.6)
**2022**										
**Month**	**Minimum daily temperature**					**Maximum daily temperature**				
	**Nas**	**Nam**	**Norm**	**Pam**	**Pas**	**Nas**	**Nam**	**Norm**	**Pam**	**Pas**
January	0 (0.7)	4 (4.0)	24 (21.7)	1 (3.9)	2 (0.7)	0 (0.2)	4 (5)	24 (21.1)	1 (3.9)	2 (0.8)
February	0 (1.0)	2 (3.1)	18 (19.5)	5 (4.0)	3 (0.4)	0 (0.7)	2 (3.9)	18 (19.1)	5 (3.8)	3 (0.5)
March	2 (0.6)	9 (4.3)	16 (20.9)	2 (4.9)	2 (0.3)	2 (0.9)	9 (4.1)	16 (21.1)	2 (4.5)	2 (0.4)
April	1 (1.2)	6 (3.6)	22 (20.6)	1 (4.3)	0 (0.3)	1 (0.4)	6 (4.9)	22 (19.6)	1 (4.8)	0 (0.3)
May	0 (0.8)	0 (4.1)	12 (21.2)	13 (4.8)	6 (0.1)	0 (0.9)	0 (4.2)	12 (21.3)	13 (4.3)	6 (0.3)
June	0 (0.7)	1 (3.8)	11 (20.9)	17 (4.2)	1 (0.4)	0 (0.9)	1 (3.8)	11 (20.4)	17 (4.7)	1 (0.2)
July	0 (0.9)	1 (4.4)	16 (20.9)	12 (4.5)	2 (0.3)	0 (1.1)	1 (3.7)	16 (21.8)	12 (4.1)	2 (0.3)
August	0 (0.8)	0 (4.3)	19 (20.7)	12 (5.0)	0 (0.2)	0 (0.9)	0 (3.6)	19 (22.3)	12 (3.7)	0 (0.5)
September	1 (0.8)	5 (4.3)	13 (20.0)	8 (4.8)	3 (0.1)	1 (0.8)	5 (3.9)	13 (20.4)	8 (4.7)	3 (0.3)
October	0 (0.7)	0 (4.1)	16 (21.4)	9 (4.6)	6 (0.2)	0 (0.7)	0 (4.1)	11 (21.3)	11 (4.3)	9 (0.6)
November	0 (0.4)	0 (4.8)	23 (19.4)	5 (5.0)	2 (0.4)	0 (0.7)	1 (3.8)	19 (21)	9 (3.9)	1 (0.6)
December	0 (0.4)	0 (4.4)	15 (21.2)	8 (4.1)	8 (0.9)	1 (0.7)	3 (4.1)	20 (21.2)	7 (4.4)	0 (0.6)

## Data Availability

Data are contained within the article and Supplementary Materials.

## Supplementary Materials

The following supporting information can be downloaded at: https://www.mdpi.com/article/10.3390/plants13081105/s1, Table S1: Statistics of Anthocyanins (Anthocyanin profile); Table S2: Chromatogram integrated at 515 nm of blueberry fruits (*Vaccinium corymbosum* L.) cv ‘Cosmopolitan’.
