# Supplementary material for: Effect of Elicitor Treatments on Quality Attributes in Blueberry: Implications of Cultivar and Environmental Conditions
Source: Plants (Basel). 2024 Apr 15;13(8):1105. doi: 10.3390/plants13081105 (PMC11054320; doi:10.3390/plants13081105)
Supplement: Supplementary file 1 [file plants-13-01105-s001.zip › Table S2.pdf]

Supplemental Table S2

Chromatogram integrated at 515 nm of blueberry fruits (*Vaccinium corymbosum* L) cv 'Cosmopolitan' containing both glycosylated anthocyanidins and acylated forms. For each detected compound is reported the retention time (RT, min), λ_{max} (nm), accurate mass of the $[M]^+$, brute formula and fragment ions $[M]^+$.

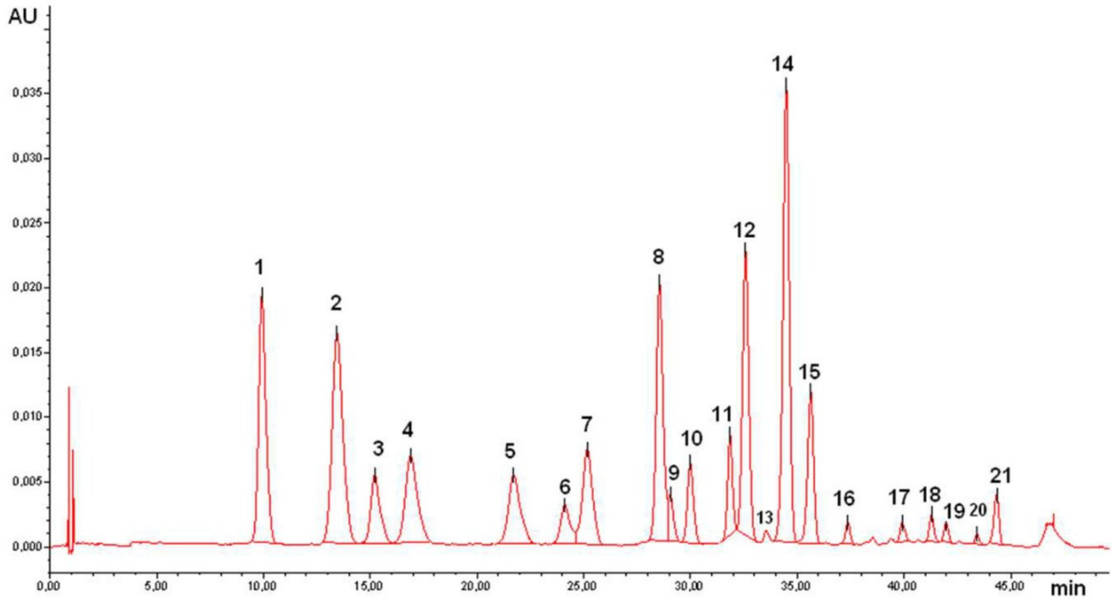

Peak	RT (min)	λ_{max}	$[M]^+$	Brute Formula	Fragment ions $[M]^+$	Brute Formula	Peak identification
1	10.0	515	465.1026	$C_{21}H_{21}O_{12}$	303.0499	$C_{15}H_{11}O_7$	D-gal
2	14.0	515	465.1026	$C_{21}H_{21}O_{12}$	303.0500	$C_{15}H_{11}O_7$	D-glc
3	15.0	515	449.1079	$C_{21}H_{21}O_{11}$	287.0548	$C_{15}H_{11}O_6$	Cy-gal
4	17.0	515	435.0920	$C_{20}H_{19}O_{11}$	303.0500	$C_{15}H_{11}O_7$	D-ara
5	22.0	515	449.1079	$C_{21}H_{21}O_{11}$	287.0548	$C_{15}H_{11}O_6$	Cy-glc
6	24.0	515	419.1000	$C_{20}H_{19}O_{10}$	287.0548	$C_{15}H_{11}O_6$	Cy-ara
7	25.0	515	479.1180	$C_{22}H_{23}O_{12}$	317.0655	$C_{16}H_{13}O_7$	Pet-gal
8	28.5	515	479.1180	$C_{21}H_{21}O_{12}$	317.0655	$C_{16}H_{13}O_7$	Pet-glc
9	29.0	515	463.1232	$C_{22}H_{23}O_{11}$	301.0705	$C_{13}H_{13}O_6$	Peo-gal
10	30.2	515	433.1127	$C_{21}H_{21}O_{11}$	317.0655	$C_{16}H_{13}O_7$	Pet-ara
11	32.0	515	463.1232	$C_{22}H_{23}O_{11}$	301.0705	$C_{13}H_{13}O_6$	Peo-glc
12	32.5	515	493.1339	$C_{23}H_{25}O_{12}$	331.0810	$C_{17}H_{15}O_7$	Mv-gal
13	33.2	515	433.1128	$C_{21}H_{21}O_{10}$	301.0705	$C_{13}H_{13}O_6$	Peo-ara
14	34.5	515	493.1339	$C_{23}H_{25}O_{12}$	331.0810	$C_{17}H_{15}O_7$	Mv-glc
15	35.4	515	463.1231	$C_{22}H_{23}O_{11}$	331.0810	$C_{17}H_{15}O_7$	Mv-ara
16	37.0	515	507.1127	$C_{23}H_{23}O_{13}$	303.0490	$C_{15}H_{11}O_7$	D-Hex-Ac
17	40.0	515	491.1180	$C_{23}H_{23}O_{12}$	287.0548	$C_{15}H_{11}O_6$	Cy-Hex-Ac
18	41.0	515	521.1280	$C_{24}H_{25}O_{13}$	317.0655	$C_{16}H_{13}O_7$	Pet-Hex-Ac
19	42.0	515	535.1444	$C_{25}H_{27}O_{13}$	331.0810	$C_{17}H_{15}O_7$	Mv-gal-Ac
20	43.4	515	505.1330	$C_{24}H_{25}O_{12}$	301.0705	$C_{13}H_{13}O_6$	Peo-Hex-Ac
21	44.5	515	535.1445	$C_{25}H_{27}O_{13}$	331.0810	$C_{17}H_{15}O_7$	Mv-glc-Ac

D: Delphinidin. Cy: Cyanidin. Pet: Petunidin. Peo: Peonidin. Mv: Malvidin. gal: galactose. glc: glucose. ara: arabinose. Hex: Hexose. Ac: acetate.
